# Muscle Spindle Alterations Following Ischemic Stroke and Peripheral Denervation in Murine Models

**DOI:** 10.3390/ijms27177826

**Published:** 2026-09-01

**Authors:** Yunfeng Sun, Xiaoxiao Zhao, Caterina Fede, Lucia Petrelli, Giulio Morri, Manuela Allegra, Andrea Porzionato, Carla Stecco

**Affiliations:** 1 Padova Neuroscience Center, University of Padova, 35129 Padova, Italy or yunfeng.sun@studenti.unipd.it (Y.S.); giulio.morri@studenti.unipd.it (G.M.); manuela.allegra@cnr.it (M.A.); 2 Institute of Human Anatomy, Department of Neuroscience, University of Padova, 35121 Padova, Italy; caterina.fede@unipd.it (C.F.); lucia.petrelli@unipd.it (L.P.); andrea.porzionato@unipd.it (A.P.); carla.stecco@unipd.it (C.S.); 3 Neuroscience Institute, National Research Council (CNR), 35131 Padova, Italy

**Keywords:** muscle spindle, proprioception, denervation, stroke, connective tissue

## Abstract

Proprioceptive dysfunction is a common rehabilitation issue in neurological injury, and the muscle spindle (MS) is considered a critical structure in this dysfunction. The present study aimed to investigate the morphological alterations of MS in two classical neurological diseases: denervation and stroke, and to understand the role of MS in these two diseases preliminarily. The denervation and stroke model animals were constructed by using a surgical operation and middle cerebral artery occlusion (MCAO) protocol, respectively. The muscles from the affected-side limb and the healthy-side limb were harvested and then fixed, embedded, sliced, stained, and subjected to other laboratory analysis at the predetermined time point. The capsule thickness, cross-sectional area (CSA) of intrafusal fiber and extrafusal fiber, the collagen area percentage, and the hyaluronic acid were investigated in the denervation and stroke animal model. In denervation rats, the capsule thickness in the denervation-side limb increased significantly with the *p* value under 0.01, while the CSA of intrafusal fiber and extrafusal fiber both decreased with the *p* values under 0.01 and 0.0001, respectively, compared to the healthy-side. The collagen area percentage increased on the denervation side, with a *p* value under 0.01, but no difference was found in hyaluronic acid. In stroke mice, similar tendencies were found in the stroke-affected side regarding the capsule thickness and CSA of intrafusal fiber, but there was no statistical significance. The CSA of extrafusal fiber seems equal on the stroke-affected side and healthy-side as well as the collagen area percentage. The hyaluronic acid in the capsule of MS was higher in affected-side compared to healthy-side with a *p* value under 0.05. The alterations of MSs suggested that MS’s roles in proprioception are diverse in these two typical neurological diseases. The changes of MS in denervation are aligned with the alterations of muscle and intramuscular connective tissue, but are not aligned in stroke, especially the hyaluronic acid. The present study provides a novel MS-based view to understand proprioception dysfunction in stroke and peripheral nerve injury.

## 1. Introduction

Muscle spindles (MSs) are specialized proprioceptive sensory organs embedded within skeletal muscles, playing a critical role in motor control, posture maintenance, and movement coordination [1]. These spindle-shaped receptors are distributed across nearly all human muscles, though their density varies depending on muscle function and species [2]. Structurally, MSs consist of intrafusal muscle fibers enclosed within a connective tissue capsule, along with afferent (sensory) and efferent (motor) nerve terminals [3]. The latest study suggested that MSs are mainly embedded in perimysium and are located at the intersection points of multiple perimysium, which come from different directions, and a small size of human muscle samples demonstrated that human MSs have a similar distribution pattern [4]. MSs function as dynamic stretch sensors, detecting changes in muscle length and velocity [4,5]. Beyond their physiological role, MSs are implicated in neuromuscular pathologies, including spasticity, motor dysfunction after stroke, and peripheral neuropathies [6,7].

Neurological diseases, including peripheral and central nerve disease, even these two conditions have diverse etiologies and pathogenesis but both have serious impacts on motor system such as the muscle fiber type, muscle morphology/components, sensor, and status of muscle due to the neurotrophic effect and the nervous reflex are impeded [8,9], among these the MS is a most common object that is affected [7,10], MS can profoundly impact motor recovery and rehabilitation strategies in above diseases, understanding the alterations of MS in central and peripheral nerve diseases is emerging as a novel concern.

However, in the last 20 years, very few studies have focused on MS alterations after neurological diseases. A previous study [11] in 2009 suggested that the peripheral denervation results in MS degeneration, both in morphology and function, while the reinnervation contributes to reversing the degeneration. Other earlier studies mainly focused on the MS-innervation after peripheral nerve injury; the evidence of MS alteration after peripheral nerve injury is not flourishing.

For most, the impacts of central nervous system diseases on MSs are not deeply investigated, especially in stroke. One study in 1999 demonstrated that the MSs rarely contribute to dysfunction in the paralyzed limb after stroke, as the spindle afferent behavior (background discharge rate, responses to reflex inputs, and responses to voluntary contractions) in the healthy-side and the paralyzed side are similar [12]. Another study [13] in 1999 also suggested that the MSs in the paralyzed limb have similar activity compare as in the healthy limb under the situation of stretching. Both these 2 studies focused on electrophysiological properties, while none had clarified the morphological alterations or others.

Above all, the MS modifications in stroke and peripheral nerve injury have not been fully investigated, especially in stroke. Acknowledging the distinct alterations of MS in each pathology, whether these alterations differ is critical for understanding the roles of MS in these disorders and developing an MS-based rehabilitation strategy in neurological diseases. In the present study, denervation and stroke animal models were used as the objects of peripheral and central nerve disease. We hypothesized that peripheral denervation and central stroke would induce distinct MS alterations, reflecting their differing neuropathological mechanisms. The main contents are as follows.

## 2. Results

### 2.1. The MSs’ Alterations in Denervation

**Figure 1:**
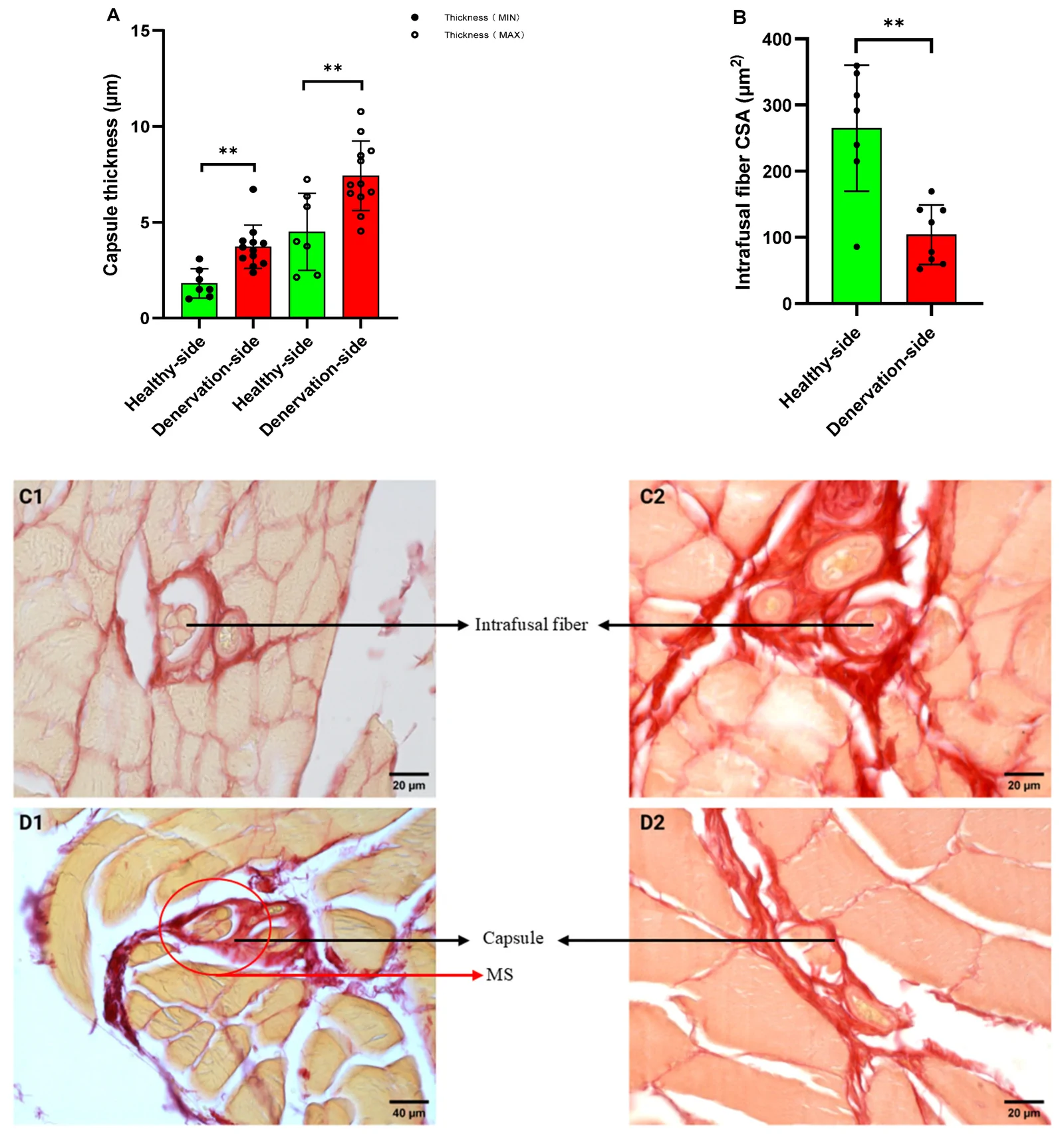
The parameters of MSs on the denervation-side and healthy-side. (**A**) The capsule thickness comparison. The healthy-side: thinnest, 1.818 ± 0.762 μm; thickest, 3.719 ± 1.126 μm. The denervation-side: thinnest, 4.503 ± 2.006 μm; thickest, 7.427 ± 1.810 μm. *t*-test, *p* = 0.0014 (thinnest); *p* = 0.0097 (thickest). (**B**) The intrafusal fiber CSA comparison. Healthy-side, 264.908 ± 95.095 μm^2^; denervation-side, 103.990 ± 44.896 μm^2^. *t*-test, *p* = 0.0014. The typical picture of MSs on the denervation-side (**C1**,**C2**) and healthy-side (**D1**,**D2**). The red circle indicates the muscle spindle (MS). Each dot represents one MS. **: *p* < 0.01.

We investigated the capsule thickness of the MSs in denervation mice. A total of 10 denervation mice were involved; 7 MSs on the healthy-side (all on the right side) were eligible for analysis, while 12 MSs on the denervation-side (all on the left side) were eligible. The thickest and thinnest parts of MSs were investigated. The thinnest and thickest in healthy-side ranged from 1.000 μm to 3.090 μm, 2.136 μm to 7.231 μm, respectively. It ranged from 2.371 μm to 6.717 μm, 4.537 μm to 10.776 μm in denervation-side. The thickness of the MS capsule increased significantly in denervation-side compare to healthy-side as shown in Figure 1A. Regarding the surface area of intrafusal fibers, the largest intrafusal fibers (nuclear bag fibers) were selected for the CSA measurements to ensure consistency, transparency, and reliability in the evaluation. Eight MSs on the denervation side were involved in the intrafusal fiber CSA analysis because the intrafusal fibers were ruined during slicing and staining in 4 of 12 MSs. The intrafusal fiber CSA ranged from 85.604 μm^2^ to 359.396 μm^2^ in healthy-side respect to 52.215 μm^2^ to 169.438 μm^2^ in denervation-side. The MS intrafusal fiber atrophied significantly under a denervation situation, as shown in Figure 1B.

### 2.2. The MSs’ Alterations in Stroke

**Figure 2:**
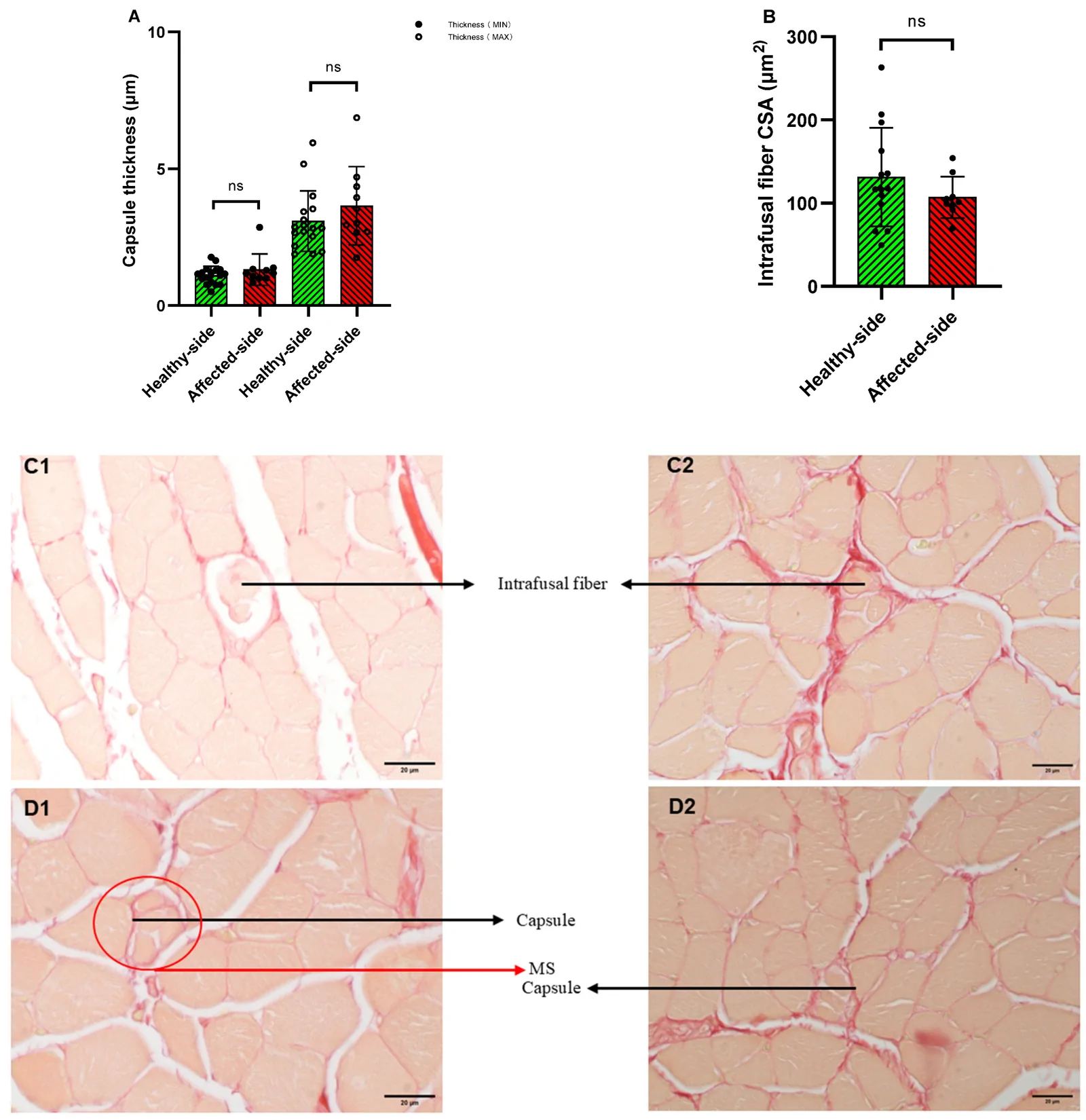
The parameters of MSs on the stroke-affected-side and healthy-side. (**A**) The capsule thickness comparison. The healthy-side: thinnest, 1.106 ± 0.323 μm; thickest, 3.089 ± 1.105 μm. The affected-side: thinnest, 1.313 ± 0.570 μm; thickest, 3.646 ± 1.432 μm. *t*-test, *p* = 0.349 (thinnest); *p* = 0.812 (thickest). (**B**) The intrafusal fiber CSA comparison. Healthy-side: 131.436 ± 59.294 μm^2^; stroke-affected-side: 107.140 ± 24.754 μm^2^. *t*-test, *p* = 0.167. The typical picture of MSs on the stroke-affected-side (**C1**,**C2**) and healthy-side (**D1**,**D2**). The scale bar in all pictures is equal to 20 μm. The red circle indicates the muscle spindle (MS). Each dot represents one MS. ns: no significance, *p* > 0.05.

The same methodology was utilized in the investigation of MSs in stroke. After MSs’ structural integrity check, 17MSs on the healthy-side and 10 MSs on the affected-side were eligible for thickness measurement, while 14 (healthy-side) and 9 (affected-side) were selected for intrafusal fiber surface area analysis. The thickness of the MS capsule on the healthy-side ranged from 0.505 μm to 1.768 μm in the thinnest region, and from 1.882 μm to 5.952 μm in the thickest region. In contrast, the thinnest regions varied between 0.833 μm and 2.862 μm, while the thickest regions ranged from 1.750 μm to 6.870 μm on the stroke affected-side. These data show an increased tendency in capsule thickness following stroke pathology, but without statistical significance, as shown in Figure 2A. The intrafusal fiber CSA in the healthy-side ranged from 49.646 μm^2^ to 262.993 μm^2^, while in the stroke affected-side was from 69.919 μm^2^ to 154.083 μm^2^. No statistical significance was found, even though the mean of intrafusal fiber CSA is less in the stroke-affected side, as shown in Figure 2B.

### 2.3. The Extrafusal Fiber Surface Area in Denervation and Stroke

**Figure 3:**
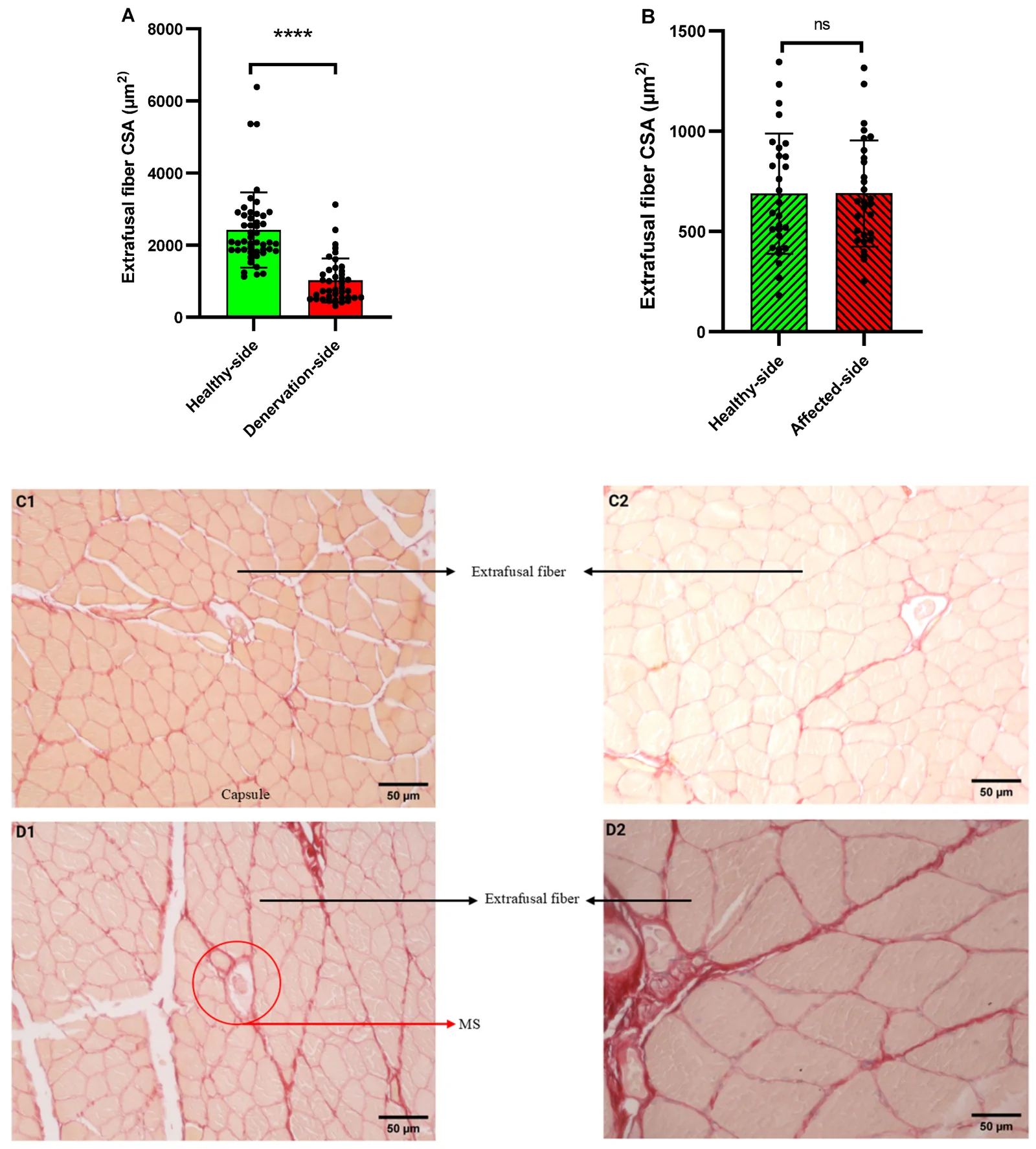
The extrafusal fiber in the denervated rat and stroke mice. (**A**) The extrafusal fiber CSA comparison in the denervated rats. The healthy-side: 2420.097 ± 1039.640 μm^2^. The denervation-affected-side: 1019.678 ± 611.130 μm^2^. *t*-test. (**B**) The extrafusal fiber CSA comparison in stroke mice. The healthy-side: 688.235 ± 300.124 μm^2^. The stroke-affected-side: 689.390 ± 265.691 μm^2^. *t*-test. The typical picture of extrafusal fibers in stroke mice ((**C1**): stroke-affected-side; (**C2**): healthy-side) and denervation rats ((**D1**): denervation-affected-side; (**D2**): healthy-side). All four pictures were in the same magnification. The yellow-stained structures represent the extrafusal muscle fibers. Each dot represents one investigated extrafusal fiber. ns: no significance, *p* > 0.05; ****: *p* < 0.0001.

The extrafusal fiber surface area is sensitive to both disorders. To understand whether the alteration of MSs’ intrafusal fiber is consistent with that of the extrafusal fiber, images were acquired using a 20× objective lens, encompassing both muscle spindles and surrounding muscle fibers in stroke mice, and the 10× objective lens was used in denervation rats following the above method. Each image was automatically divided into a 3 × 3 grid (nine equal subregions) using a custom macro in FIJI (windows-x64), which also randomly selected two subregions for analysis. In each selected subregion, all clearly identifiable and morphologically intact extrafusal muscle fibers were manually outlined and their CSA was measured using FIJIJ. All measured extrafusal fibers’ CSAs are shown in Figure 3.

### 2.4. The Collagen Area Percentage in Denervation and Stroke

**Figure 4:**
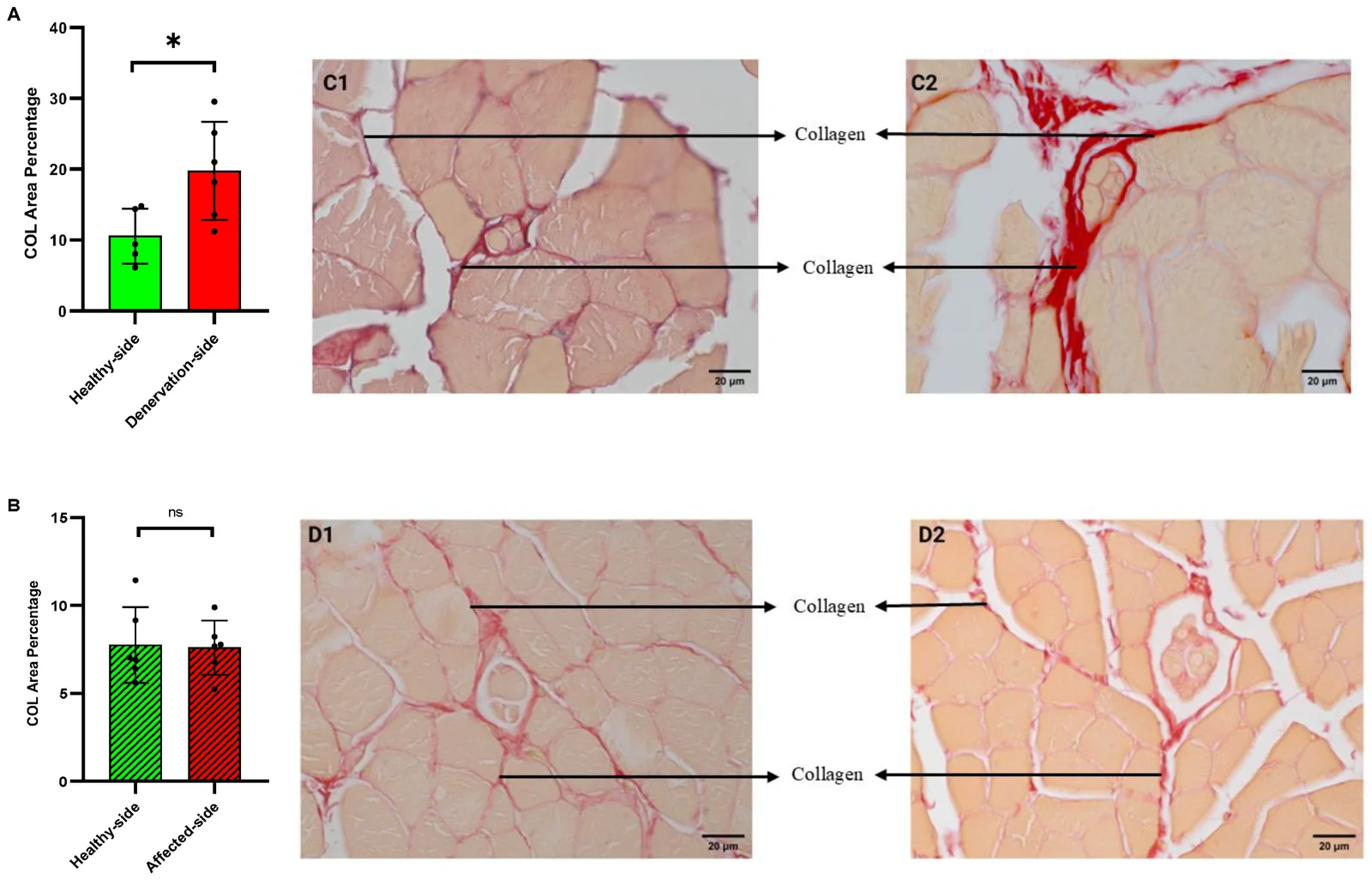
The collagen alteration in denervation and stroke. (**A**) The collagen area percentage in denervation (%, healthy-side: 10.56 ± 3.86, *n* = 5; six pictures were selected randomly, and one was excluded due to containing a vessel in this section; affected-side: 19.77 ± 6.92, *n* = 6); *t*-test, *p* = 0.0269. (**B**) The collagen area percentage in stroke (%, healthy-side: 7.76 ± 2.16, *n* = 6; affected-side: 7.61 ± 1.55, *n* = 6). (**C1**,**C2**) The typical Sirius Red staining sections showed the increased collagen area percentage in denervation rats ((**C1**): The healthy-side, (**C2**): The denervation-side). (**D1**,**D2**) The typical Sirius Red staining sections showed the increased collagen area percentage in stroke mice ((**D1**): the healthy-side, (**D2**): the affected-side). COL: collagen, stained red in the figures. Each dot represents one randomly selected MS, and the microscopic image is centered on that MS. ns: no significance, *p* > 0.05; *: *p* < 0.05.

The above results suggested that the thickness of MS’s capsule increased significantly in denervation rats, while there was no significance in stroke mice, even though the average value increased slightly. Our previous study had confirmed that the MS is embedded in perimysium mainly [4]; assessing the alterations of collagen area percentage around MSs greatly contributes to understanding the modifications of MSs comprehensively. In denervation, the collagen area percentage ranges from 11.23% to 29.52% on the denervation-side, from 6.13% to 14.78% on the healthy-side. Collagen area percentage in denervation-side increased significantly, as shown in Figure 4A,C. Regarding the stroke mice, the area percentage of collagen has no difference between healthy-side (5.60% to 11.43%) and the affected side (5.23% to 9.90%), as shown in Figure 4B,D.

### 2.5. The Alterations of HA in the MSs’ Capsule in Stroke and Denervation

**Figure 5:**
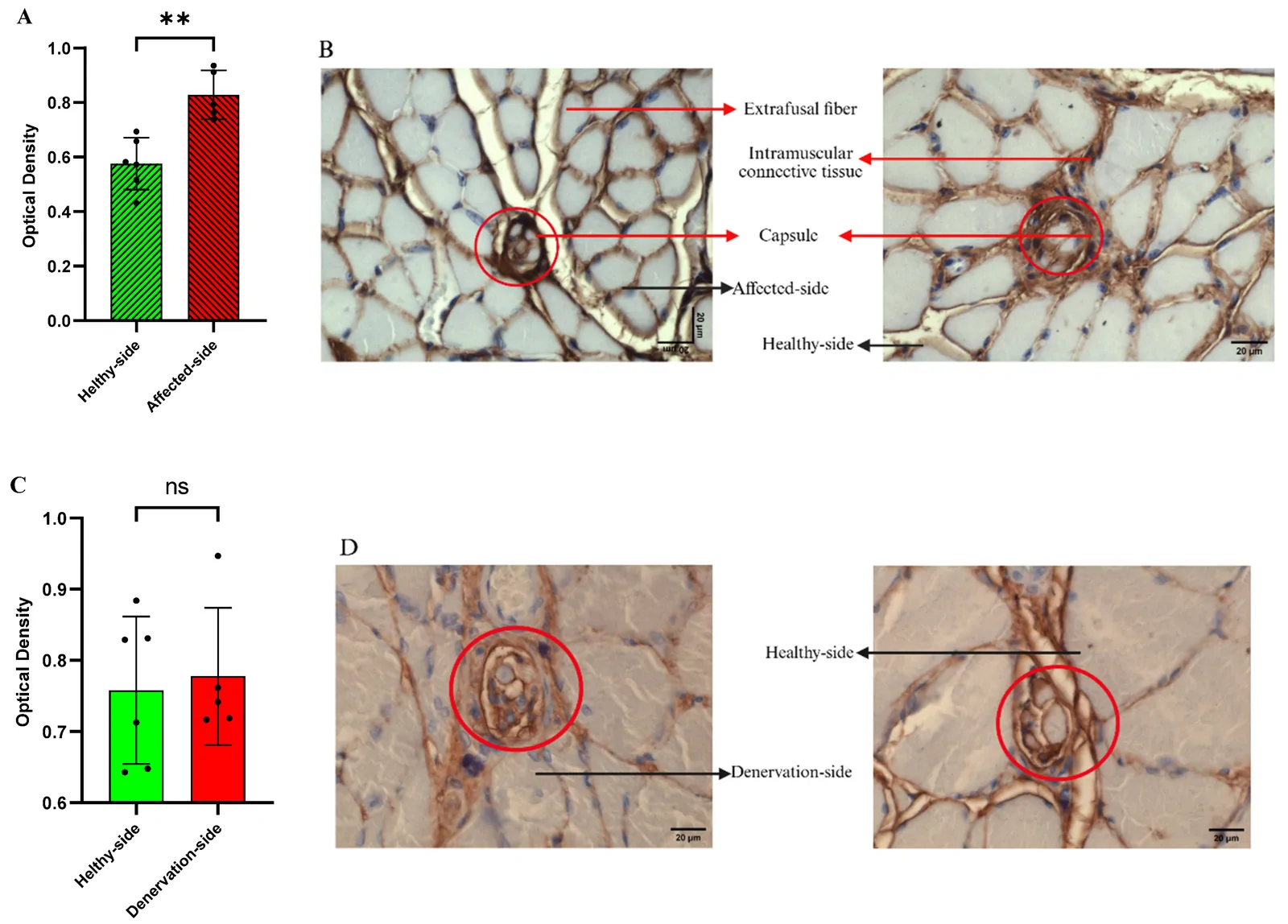
The HA alterations in stroke mice and denervation rats. (**A**,**B**) In stroke mice, the OD value on the affected-side was 0.83 ± 0.080 (*n* = 5) and on the healthy-side was 0.58 ± 0.087 (*n* = 6). Unpaired *t*-test, *p* = 0.015. The typical IHC picture was shown in (**B**). (**C**,**D**) In denervation rats, the OD value on the affected-side was 0.78 ± 0.087 (*n* = 5) and on the healthy-side was 0.76 ± 0.095 (*n* = 6). The *t*-test, *p* = 0.757. The typical IHC picture was shown in (**D**). The red circles represented the MSs, and the brown color stands for the HA. **: *p* < 0.01.

The HA was examined by IHC; the optical density (OD) value is utilized to present the situation of HA because the quantitative analysis of HA in MSs’ capsules is not feasible. Regarding the stroke mice, the OD level of HA is significantly higher on the affected-side (OD: 0.74–0.94) than on the healthy-side (OD: 0.43–0.69), as shown in Figure 5A,B. However, there is no difference between the denervation-side and healthy-side in denervation rats, as shown in Figure 5C,D; the OD value in the denervation-side ranges from 0.72 to 0.95, while the value ranges from 0.64 to 0.88.

## 3. Discussion

The application value of MSs in the above disorders is limited, as the alterations of MSs in nerve system disorders are not fully understood [3,14]. The present study aims to investigate the morphological alterations of the muscle spindle based on two classical nerve disease model animals (peripheral nerve injury: sciatic nerve denervation rats, and central nervous system disease: stroke mice) to present a preliminary understanding of the MS alterations in these two pathologies.

There is a lack of studies focused on MS alterations in stroke; two earlier studies suggested that the neuroactivity of MSs in the affected limb is similar to the healthy-side based on surface electromyogram (EMG) [12,13], but the alterations of MS were not investigated. Regarding denervation, one investigation [11] illustrated that the diameter of MSs reduced after peripheral nerve injury, but the characteristics of the capsule and intrafusal fiber were not evaluated. Other studies [15,16] mainly aimed at the MSs’ neuronal functions after nerve injury and reinnervation. The morphological/structural alterations of MSs after nerve injury (central and peripheral) are overlooked. The present study provides novel firm evidence to address the gap in MS morphological alterations in classical nerve injury disorders.

In the denervation rats, a significant increasing of capsule thickness was observed, as well as the related intramuscular connective tissue, and the data demonstrated that the capsule thickness increased significantly, as well as the collagen area percentage around the MSs. The results align with the previous studies: the total collagen content and collagen area percentage both increased in the denervation rat [17]. The increased area percentage of collagen may be related to two factors: Attributing to the reduced muscle volume and myofiber atrophy; An absolute increase in collagen content, which has been confirmed by the study [17]. The increasing of connective tissue is considered an adaptive change after peripheral nerve injury: a previous study [18] confirmed that expression of connective tissue growth factor (CTGF) was up-regulated after sciatic nerve injury and contributed to the fibrosis; another study [19] also suggested that the fibrosis of connective tissue exists in carpal tunnel syndrome. Our previous study [4] had confirmed that the MSs are embedded in perimysium and dominantly locate in the intersection of perimysium, the aforementioned anatomical relationships offer a plausible explanation for the near-synchronous occurrence of changes in MS capsule thickness and the morphology of intramuscular connective tissue, above relationship between MS and connective tissue (perimysium) also suggested that the alterations of MS’s capsule and the connective tissue around MS may affect the activity of MSs, but further neurological or EMG investigations are needed to address this issue. The CSA of MS intrafusal fiber and extrafusal muscle fiber both decreased significantly compared to the healthy-side; the results may be considered a typical pathology of atrophy after peripheral nerve injury [20].

The capsule thickness, collagen content/collagen area percentage, and intrafusal fiber exhibited significant alterations following denervation; the respective effects of these alterations on MSs’ function are likely distinct and should therefore be analyzed separately. First, whether the intrafusal fiber CSA degeneration will contribute to the dysfunction of MSs remains questionable. Intrafusal fibers function like springs, serving as mechanical carriers during stretch-related activity [3]. Through afferent innervation, they convert the degree of elongation or shortening into electrical signals [21]. However, whether a reduction in their cross-sectional area leads to abnormal stretch or contraction responses remains highly questionable; this assumption lacks a solid anatomical foundation [3,5]. However, alterations in the capsule and intramuscular connective tissue may have greater implications for muscle spindle (MS) function and, therefore, warrant more in-depth investigation. The collagen-made capsule plays as a tube and container permits the gliding and activity of intrafusal fiber from the anatomical view, studies [22,23] have confirmed that excessive collagen fiber and the alterations of components of fascia strongly leads to the stiffness of the tissue, imped the inter-activity between connective tissue and the around structure, for instance, the muscle fiber gliding, and the connective tissue lost the ability which to adapt the muscle fiber volume change.

In stroke mice, a trend toward increased capsule thickness and decreased intrafusal fiber size was observed in affected-side. However, neither change reached statistical significance. In contrast, the extrafusal fiber size showed minimal variation between the affected and the healthy-side as well as the collagen area percentage around the MSs. These results raise the hypothesis that alterations of MSs may occur earlier than changes in muscle fibers and intramuscular connective tissue. Previous work demonstrated that the onset timing of muscle size alteration varies, ranging from one week to months, and is strongly related to the activities and rehabilitation after stroke [24]. A study from Xu J, et al. suggested that the proprioception dysfunction after stroke decreases linearly over time after stroke [25]. These conclusions revealed that there is no certain correlation between proprioception dysfunction and alterations of extrafusal muscle fibers in stroke. Combining with the previous study [4], several possibilities and hypothesis are concluded according the results of present study: The earlier change of MSs such as capsule thickness possible contributes to the proprioception dysfunction as an independent factor in stroke, the increasing in the thickness of the capsule affects the sliding and sensing capabilities of the intrafusal fiber within the MSs; The alterations of MS have no roles in proprioception dysfunction in stroke; The MSs play roles in stroke but the onset timing is not defined. To address the above issues, further studies are urgently needed to demonstrate the relationship between capsule/intrafusal fiber/connective tissue and proprioception, especially the time-based and potentially event-related relationship.

Regarding the above differences in MSs’ alterations between peripheral nerve injury and stroke, the “sensory protection” mechanism is considered to be the key factor to explain the above results [11,26]. A previous study has suggested that embryonic dorsal root ganglion cell transplantation helps to maintain the structural and functional integrity of muscle structures and muscle spindles in denervated injuries, which points out that the afferent nerve is a key factor in maintaining the function of MSs [27]. Studies in neonatal rats have confirmed that group Ia sensory fibers promote muscle spindle differentiation and suppress the degeneration of MSs. Nerve growth factor (NGF) can retain this function by promoting the growth of group Ia sensory fibers in a denervated state [28]. Another study in mice showed that degeneration of Ia axons innervating the spindle was significantly reduced when NGF neurotrophic factor-3 was overexpressed, and by improving Ia-spindle interactions, group II and γ fibers were also improved after injury [16]. The above evidence suggests that the structure of MSs is mainly related to the integrity of sensory fibers, suggesting that the changes in MSs during sciatic injury in the affected side limb in this study may be partially due to sensory fiber damage. The above evidence also serves as a potential mechanism to explain the reason for MSs’ similarity between the stroke-affected limb and the unaffected limb; the integrity of sensory fibers may contribute to maintaining the structure of MSs. The alternations of MSs are reversible in peripheral nerve injury when proper repair is utilized, study of Kenichi Asano, et.al [27] demonstrated that the quantity and morphological characteristics of MSs following tibia nerve injury were improved simultaneously after the intervention of transplanted embryonic dorsal root ganglion cells, compared to surgery group, but the MSs in the group of transplanted embryonic dorsal root ganglion cells were not fully similar with the MSs in normal control group. Most importantly, study of Doherty C suggested that recovery ability is strongly related to earlier repair treatments after peripheral nerve injury, especially the repair of the sensory nerve [29].

Interestingly, the HA alteration in stroke and denervation is totally inconsistent. HA increased in the affected-side MSs compared to the healthy-side in stroke mice, while no difference was found in denervation rats. The HA alteration in stroke has been investigated in a few studies: The previous study [30] confirmed that the HA increased in the plasma/brain of stroke patients; further investigation suggested that low-molecular-weight HA is the main type of HA that increased, as well as hyaluronidase. Increased hyaluronidase activity results in the breakdown of high-molecular-weight HA. Regarding the mechanisms, study [30] suggested that the inflammation after stroke may contribute to the alteration of HA in stroke; However, the relationship between HA concentration and prognosis is complex and unclear to date [31]. But above studies can’t explain the results of present study regarding the HA alterations of MS in stroke mice (increased in stroke affected-side, mainly concentrated in capsule of MS and intramuscular connective tissue), this alteration may relate to the changes of intramuscular connective tissue, but does not due to the systemic inflammatory response or its association with the elevated levels of HA in the plasma which were mentioned in other studies. Most importantly, whether this alteration will modify the function of MSs is not estimated, and no studies have focused on this to date. Further studies are needed to investigate the HA and muscle spindles. HA plays vital role in maintaining the proper biomechanical characteristics of fascia, such as gliding, force transmission, and tissue viscosity [32]. Our previous study [4] suggested that the above characteristics may affect MS function due to the anatomical relationship between fascia and MSs. Rehabilitation plays an important role in maintaining and improving proprioception after stroke [33], potentially through exercise-induced modulation of the biological properties of extracellular matrix components, such as HA, thereby optimizing the biomechanical environment surrounding MSs and facilitating the recovery of proprioceptive function based on the results of the present study. However, this proposed mechanism remains hypothetical and requires further investigation. HA-based treatment represents a potential strategy to regulate MS function and status in stroke.

## 4. Limitations

Limitations exist in the present study. First, the investigation of morphological alterations is not sufficient to determine the accurate roles of MSs in proprioception dysfunction in the above diseases; the potential events are considered a critical supplementary method that should be examined in the future. Second, this study examined the morphological changes of MSs at a specific time point after stroke in mice and did not detect significant differences. However, such changes may be time-dependent. Due to the lack of longitudinal or time-point-stratified analysis, the current findings are insufficient to determine the dynamic alterations of MSs following stroke, which represents another limitation of this study; this will be addressed in a future study.

## 5. Materials and Methods

### 5.1. Animal Model Construction

The denervation animal model was established using identical procedures to our previously published protocol as follows: Twelve Sprague–Dawley rats (8 weeks old) were used in this study. A sciatic nerve denervation model was established as previously described by our group [17]. Experimental procedures were approved by the ethical committee of the University of Padua (No. 837/2019-PR, 9 December 2019). Briefly, animals were anesthetized with isoflurane and oxygen, and the left sciatic nerve was exposed through a gluteal-splitting approach and transected to induce denervation. Following surgery, the incision was closed in layers, and animals were allowed to recover under standard housing conditions. Postoperative care and animal monitoring were performed in accordance with the previously published protocol. A sciatic function index score of −87 ± 9.12 was observed at 6 weeks after surgery [17]; the denervation animal model was successfully constructed. For the stroke mice, right middle cerebral artery occlusion (MCAO) was used to construct the model, the experiments were approved by the Italian Ministry of Health (nr 935/2024-PR) and the details of the methods were described in a previous study [34]: Eleven 8-week-old C57BL mice were involved in the present study as stroke mice model; the left side limb was the stroke-affected side, while the right side was the healthy side. All mice underwent the grid walk test at baseline (Day 0) and 30 days after MCAO. In brief, the total number of steps (both sides) and foot faults (the stroke-affected-side paw) were counted, and the foot fault percentage was calculated using the formula as described in the previous study [34]. The Day 0 baseline value for each mouse was normalized to 1, and the Day 30 results are presented as the change relative to the individual baseline. The mean of Day 30 was 1.6844 ± 0.6944 (paired *t*-test, *p* = 0.0084), indicating locomotor deficits in the stroke mice.

### 5.2. Tissue Harvesting and Processing

Denervation rats: Six weeks after surgery, all rats were euthanized by carbon dioxide (CO_2_) asphyxiation, and the gastrocnemius muscles from the denervated and contralateral healthy sides were harvested. Stroke mice: Mice were euthanized by intraperitoneal injection of pentobarbital 30 days after surgery, and muscles in the entire distal part of the forelimb of both sides were harvested. Then, the fixation and embedding procedures were followed. Muscle segments were immersion-fixed in 10% neutral buffered formalin for 24 h to preserve structural integrity. Following fixation, tissues underwent systematic dehydration through an ascending ethanol gradient (70%, 80%, 95%, and absolute ethanol) before being cleared in xylene. Although alternative clearing agents were evaluated, xylene was retained due to its optimal paraffin compatibility.

For paraffin infiltration, tissues were immersed in molten wax (56–60 °C) under vacuum conditions to enhance penetration, with two infiltration cycles performed to ensure thorough saturation. To minimize tissue distortion, temperature regulation was strictly maintained during this process. Embedding was conducted using pre-warmed molds filled with paraffin, with specimens strategically oriented to facilitate transverse fiber sectioning. Rapid solidification was achieved by cooling on a chilled plate, ensuring optimal block stability for microtomy.

### 5.3. Sectioning and Slide Preparation

Sections (5 µm thickness) were obtained using a Leica RM2255 automated rotary microtome (Leica Biosystems Italia, Buccinasco, MI, Italy), with 3–4 non-consecutive sections collected per sample. To prevent artifacts, blades were replaced periodically, and section quality was verified via stereomicroscopic examination. Sections were mounted on glass slides and dried overnight at 37 °C to enhance adhesion before staining.

### 5.4. Histochemical Staining and Analysis

Sirius Red staining (0.1% in picric acid, 15 min) was employed to delineate MS morphology, muscle fibers, and connective tissue, with connective tissue staining red and muscle fibers appearing yellow. MS identification relied on key histological features: the presence of encapsulated intrafusal fibers, notably thinner than extrafusal fibers. To ensure comprehensive MS detection, entire sections were systematically scanned in a linear fashion rather than by random sampling. All MSs that were identified from all sections were recorded to investigate the parameters (capsule thickness, intrafusal fiber surface area) of MSs.

Regarding the extrafusal muscle fiber cross-sectional area (CSA) analysis, the random strategy was selected: Sections where the structures were not ruined were selected. The most intact section was selected from each muscle specimen of the limb. After this, the FIJI software (windows-x64) was utilized to divide each section into 9 equally sized areas (3 × 3 grid). Two areas were selected randomly by FIJI software to avoid positional bias (the Java code was submitted as a complementary file). Exclusion criteria: areas with artifacts (folds, tears, vessels, nerve fibers, epimysium, or poor staining) were excluded, and replacement areas were reselected randomly (maximum 2 attempts) if these 2 areas were both not qualified. The results (extrafusal fiber CSA and collagen fiber percentage) of the two areas were used for statistical analysis.

The collagen percentage analysis. From both the denervation and stroke animal models, 6 spindles were randomly selected from the affected side and 6 from the healthy side in each model by using Random.org. For each selected muscle spindle, an image was acquired centered on the spindle using a 40× objective. Images containing large blood vessels were excluded due to the vascular walls containing dense collagen-rich extracellular matrix that would artificially elevate the Sirius Red–positive area. Collagen quantification was performed using Fiji. The Sirius Red signal was isolated using the standard Sirius Red color-deconvolution matrix. The collagen area fraction was calculated as: “Collagen fraction” = “collagen-positive area”/”total ROI area (40× field)”. Microscopic imaging was performed using Leica DMR and K3C systems, with subsequent morphometric analysis (MS, surface area, and collagen percentage) conducted via FIJI software.

### 5.5. Hyaluronic Acid (HA) Analysis

Immunohistochemistry (IHC) was employed to investigate the level of HA. Paraffin-embedded tissue sections were deparaffinized and rehydrated using standard procedures. Endogenous peroxidase activity was blocked with 1.5% hydrogen peroxide (in PBS) for 10 min. After washing with PBS, sections were blocked with 0.2% bovine serum albumin for 1 h in PBS, then treated with hyaluronic acid binding protein (HABP, 1:1000) and maintained overnight at 4 °C. After washing 3 times with PBS, the sections were incubated with HRP-conjugated Streptavidin for HABP (1:250) for 30 min at room temperature. Following repeated washings with PBS, the reaction was developed with 3,3′-diaminobenzidine. The negative controls were obtained by omitting the primary antibodies. Each slide was followed by the procedures of H&E stain, then was dehydrated in a graded ethanol series and mounted for microscopic evaluation. Images were acquired under identical conditions, and staining intensity was quantified by optical density analysis using FIJI.

### 5.6. Statistical Evaluation

Data were processed using PRISM 8.0.2 (GraphPad). All results were presented as mean ± SD. Two-group comparison utilized Student’s *t*-test, with statistical significance defined as *p* < 0.05. For continuous variables, the *t*-test was used when the data were normally distributed and variances were homogeneous. When variances were unequal, Welch’s *t*-test was applied. For non-normally distributed data, non-parametric tests were used.

## 6. Conclusions

The present study demonstrated the MSs’ alterations in two classical diseases: Denervation and stroke. The capsule thickness increased while the CSA of intrafusal fiber decreased significantly in the denervation-affected side compared to the healthy-side; In stroke mice, a similar tendency was observed, but both the thickness of the capsule and the CSA of intrafusal fiber had no significance. These results suggested that the alterations of MSs play a critical role in proprioception dysfunction after denervation, combining with other studies’ conclusions about the connective tissue alteration and the interplay between connective tissue and surrounding muscle fiber, but further neuro-electrophysiology was suggested to confirm the above conclusion. Regarding the MSs in stroke, the morphological alterations were still not defined in the present study, as well as the roles of MSs in proprioception dysfunction, even though the HA was modified significantly between the stroke-affected side and healthy-side. Longitudinal studies analyzing MSs at multiple post-stroke time points, combined with neuro-electrophysiological techniques, would provide a more comprehensive understanding of their dynamic changes and help to elucidate their role in stroke pathology and recovery.

## Data Availability

Data are available from the corresponding author upon reasonable request.
